# Using Twitter to Surveil the Opioid Epidemic in North Carolina: An Exploratory Study

**DOI:** 10.2196/17574

**Published:** 2020-06-24

**Authors:** Mohd Anwar, Dalia Khoury, Arnie P Aldridge, Stephanie J Parker, Kevin P Conway

**Affiliations:** 1 North Carolina A&T State University Greensboro, NC United States; 2 Research Triangle Institute International Research Triangle Park, NC United States

**Keywords:** opioids, surveillance, social media

## Abstract

**Background:**

Over the last two decades, deaths associated with opioids have escalated in number and geographic spread, impacting more and more individuals, families, and communities. Reflecting on the shifting nature of the opioid overdose crisis, Dasgupta, Beletsky, and Ciccarone offer a triphasic framework to explain that opioid overdose deaths (OODs) shifted from prescription opioids for pain (beginning in 2000), to heroin (2010 to 2015), and then to synthetic opioids (beginning in 2013). Given the rapidly shifting nature of OODs, timelier surveillance data are critical to inform strategies that combat the opioid crisis. Using easily accessible and near real-time social media data to improve public health surveillance efforts related to the opioid crisis is a promising area of research.

**Objective:**

This study explored the potential of using Twitter data to monitor the opioid epidemic. Specifically, this study investigated the extent to which the content of opioid-related tweets corresponds with the triphasic nature of the opioid crisis and correlates with OODs in North Carolina between 2009 and 2017.

**Methods:**

Opioid-related Twitter posts were obtained using Crimson Hexagon, and were classified as relating to prescription opioids, heroin, and synthetic opioids using natural language processing. This process resulted in a corpus of 100,777 posts consisting of tweets, retweets, mentions, and replies. Using a random sample of 10,000 posts from the corpus, we identified opioid-related terms by analyzing word frequency for each year. OODs were obtained from the Multiple Cause of Death database from the Centers for Disease Control and Prevention Wide-ranging Online Data for Epidemiologic Research (CDC WONDER). Least squares regression and Granger tests compared patterns of opioid-related posts with OODs.

**Results:**

The pattern of tweets related to prescription opioids, heroin, and synthetic opioids resembled the triphasic nature of OODs. For prescription opioids, tweet counts and OODs were statistically unrelated. Tweets mentioning heroin and synthetic opioids were significantly associated with heroin OODs and synthetic OODs in the same year (*P*=.01 and *P*<.001, respectively), as well as in the following year (*P*=.03 and *P*=.01, respectively). Moreover, heroin tweets in a given year predicted heroin deaths better than lagged heroin OODs alone (*P*=.03).

**Conclusions:**

Findings support using Twitter data as a timely indicator of opioid overdose mortality, especially for heroin.

## Introduction

Opioid overdose deaths (OODs) constitute a significant public health burden for the United States. In 2018, of the 67,367 drug overdose–related deaths, 70% (46,802) were attributed to opioids, with increases across demographic and geographic subgroups. Additionally, OODs involving synthetic opioids (eg, fentanyl) increased 10% from 2017 to 2018 and accounted for two-thirds of opioid-related deaths [1]. By contrast, rates of OODs involving heroin and prescription opioids decreased between 2017 and 2018 (by 4.1% and 13.5%, respectively).

Reflecting on the evolving nature of the opioid crisis, Dasgupta, Beletsky, and Ciccarone [2] present an explanatory triphasic framework. The first phase, beginning in 2000, was based on prescription opioids for pain. The second involved a sharp increase in heroin overdose deaths between 2010 and 2015. The third phase saw a rapid increase in overdose deaths attributable to synthetic opioids, beginning in 2013.

Currently, the monitoring of OODs relies primarily on mortality data that lag between 12 to 18 months behind real time. Given the rapidly shifting nature of OODs, timelier surveillance data are critical to inform strategies that combat the opioid crisis. Over the last several years, there have been over 1000 health-related publications using Twitter to inform health research. This body of science spans a number of disparate areas, including tracking the spread of influenza [3,4], oral health problems [5], sleep issues [6], obesity [7], cardiovascular disease [8], diabetes [9], mental health [10], and health care enrollment [11]. In addition, there is burgeoning interest in the use of innovative and nontraditional methods (such as mining and analyzing social media data) as a means to better surveil the opioid epidemic, with Twitter becoming a complementary data source for pharmacovigilance [12,13].

Regarding opioids specifically, researchers have analyzed Twitter messages and other social media posts from forums such as Reddit to understand their role in recovery from opioid use disorder [14], and access to and diversion of prescription drugs [15-18] and illicit opioids [19]. Twitter data have also been mined to study perceptions and attitudes toward opioids [20-22], including those held by specific groups such as youth [23]. Researchers have used other data streams, including Google Trends to forecast premature death from alcohol, drugs, and suicides [24]; a cryptomarket forum on the Dark Web to assess the emergence of new psychoactive substances [25]; and WebMD to explore motivations to use buprenorphine [26,27]. Recently, Graves et al [28] reported that thematic patterns of opioid-related tweets correlated with opioid overdose rates at the state and county levels. Sarker et al [29] reported that opioid-related tweets in Pennsylvania correlated with county-level OODs over 3 years. However, no study investigated whether opioid-related tweets in a given year can predict subsequent OODs.

This study explored Twitter data to monitor the opioid epidemic. Specifically, this study investigated the extent to which the content of opioid-related tweets corresponds with the triphasic nature of the opioid crisis and correlates with OODs in North Carolina between 2009 and 2017. North Carolina was selected because of its high rates of OODs, which increased notably during the study period.

## Methods

Data collection from Twitter involved retrospectively monitoring the platform using Crimson Hexagon to access all English opioid-related posts from January 1, 2009, through December 31, 2018, in North Carolina. We created queries (opinion monitors) with a set of parameters (search terms) in Crimson Hexagon including commercial (eg, oxycodone, codeine, and morphine) and “street” names (eg, white, syrup, and tar) of drugs. We cast a broad net to capture terms referencing both trade and generic names. In order to identify such terms, we searched for common slang words referring to opioids using the Drug Enforcement Administration’s (DEA) Intelligence Report titled “Slang Terms and Code Words: A Reference for Law Enforcement Personnel” [30]. We subsequently eliminated posts in which the slang term (eg, “China”) appeared without any mention of the identified search term parameters elsewhere in that post. We excluded posts that contained hyperlinks as well as those containing solicitation-related words such as “buy” and “sell” as these were likely to be related to illegal online drug promotion or spamming techniques encouraging users to link to other sites.

Post location was determined through cross-verification of the geotag, profile information, time zones, content, and image data. This process resulted in a corpus of 100,777 posts consisting of tweets, retweets, mentions, and replies. We made the decision not to exclude retweets with the understanding that retweets signify a unique form of communication through an implied endorsement or agreement with the initial post [31].

Using a random sample of 10,000 posts from the corpus, we identified opioid-related terms by analyzing word frequency for each year. Next, we coded these terms into three tweet categories: prescription opioids (eg, codeine, morphine, pain, hydrocodone, pills, syrup, oxycodone, oxycontin, Percocet, and Vicodin), heroin (eg, heroin, tar, and white), and synthetic opioids (eg, fentanyl, synthetic, and laced).

We obtained annual mortality data from 2009 to 2018 from the Multiple Cause of Death database from the Centers for Disease Control and Prevention Wide-ranging Online Data for Epidemiologic Research (CDC WONDER) [32]. Drug overdose deaths were classified using the 10th revision of International Classification of Diseases (ICD-10), based on the ICD-10 underlying cause-of-death codes X40-X44 (unintentional), X60-X64 (suicide), X85 (homicide), or Y10-Y14 (undetermined intent). Drug overdoses with the following codes were considered OODs: opium (T40.0), heroin (T40.1), natural and semisynthetic opioids (T40.2), methadone (T40.3), synthetic opioids other than methadone (T40.4), and other unspecified narcotics (T40.6).

We estimated the association between the opioid-related tweet categories and OODs using ordinary least squares regression with either the current tweet count or a 1-year lag of tweet count as the independent variable. We also fit a vector autoregression and used Granger tests [33] to determine whether lagged tweet counts predict OODs better than lagged OODs alone. Stationarity for each of the six series was tested using an augmented Dickey-Fuller [34] unit root test with up to two lags and a linear trend. Analyses used Stata/MP (Version 15.1; StataCorp LLC).

This study consisted of secondary analyses; no individuals were involved. As data do not include any personally identifiable information, Institutional Review Board approval was not required.

## Results

**Figure 1:**
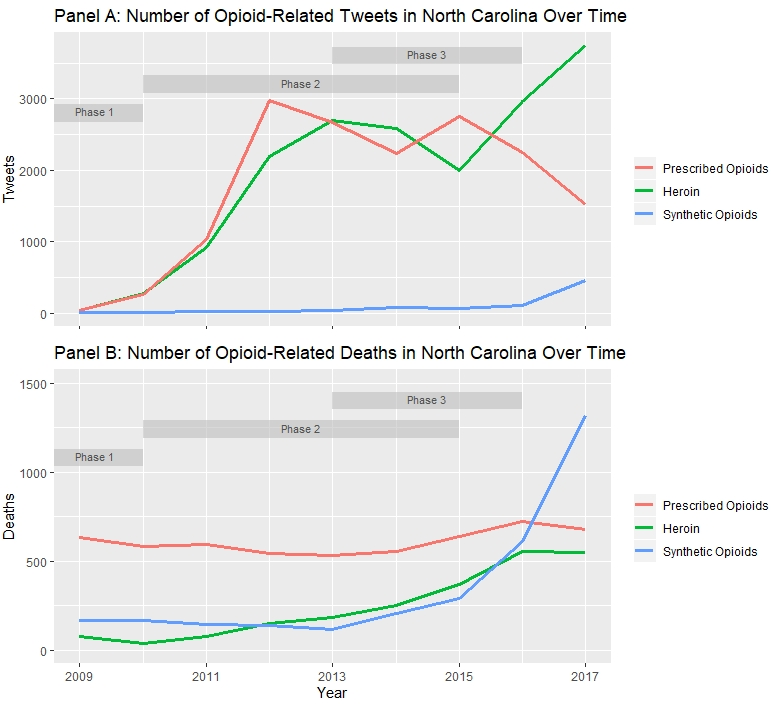
Pattern of opioid-related deaths and tweets in North Carolina over time.

The pattern of opioid-related Twitter posts in North Carolina appears in Figure 1A. Tweets about prescription opioids and heroin progressed in a similar, nonlinear pattern until they diverged in 2015, when heroin tweets increased and tweets for prescription opioids decreased. Tweets about synthetic opioids were virtually nonexistent until 2016, when they increased.

The progression of OODs in North Carolina appears in Figure 1B. Prescription opioids were the leading cause of OODs from 2009 to 2016. Heroin was the third leading cause of OODs until 2012, the second leading cause until 2016, and the third leading cause in 2017. Fentanyl became the leading cause of OODs in 2017.

Using the augmented Dickey-Fuller tests, we failed to reject stationarity up to two lags for all variables except for prescription OODs. The association between tweet count and OODs was not significant for prescription opioids in either the one-year lag model (coefficient=0.01; *P*=.58) or the no-lag model (coefficient=0.01; *P*=.64). In contrast, tweet counts for both heroin and synthetic opioids were significantly associated with OODs. On average, each additional heroin tweet in a given year corresponded to 0.13 additional heroin overdose deaths that same year (*P*=.01) and 0.13 additional deaths the following year (*P*=.03). Each additional tweet mentioning synthetic opioids in a given year corresponded to 2.68 additional synthetic opioid overdose deaths that year (*P*<.001) and 9.24 additional deaths the next year (*P*=.01).

Granger tests following vector autoregression estimation with one and two lags (only one lag was estimated for prescription OODs) were consistent with the regression results but significant only for heroin tweets; tweets mentioning heroin in a given year significantly predicted subsequent heroin OODs (*P*=.03) over and above lagged heroin OODs.

## Discussion

The pattern of opioid-related Twitter posts in North Carolina resembled the triphasic nature of the opioid crisis as described by Dasgupta et al [2]. Tweets about prescription opioids and heroin were intertwined through the end of Phase 2, when tweets about prescription opioids declined and tweets about heroin surged. During Phase 3, tweets about synthetic opioids emerged around 2016.

Results from the regression models and Granger tests indicated that the association with OODs differed by the type of opioid. For prescription opioids, tweet count and OODs were unrelated. The lack of association observed between prescription opioid tweets and overdose deaths may underestimate the true association therein, particularly because some patients who are treated with prescription opioids are chronic pain patients or older individuals [35], who may be less likely to have an active presence on Twitter. Indeed, almost half of Twitter users are aged 18 to 24 years (44%), followed by those aged 25 to 29 years (31%), 30 to 49 years (26%), 50 to 64 years (17%), and ≥65 years (7%) [36]. Although our sample may underestimate the association among individuals aged 50 years and older, this bias seems likely to be minimal because the majority of opioid overdose–related deaths in 2018 occurred among individuals aged 25 to 44 years [1].

Tweets mentioning heroin and synthetic opioids were significantly associated with heroin OODs and synthetic OODs, respectively. Moreover, results from the Granger tests showed that heroin tweets in a given year predicted subsequent heroin deaths better than lagged heroin OODs alone. These predictive results extend recent reports of correlations between opioid-related tweets and opioid overdose rates at the state and county levels [28,29].

There are a number of limitations to be considered. First, the scope of the terms used in our search parameters was somewhat subjective, in that there are hundreds of terms representing opioids [30], and we selected the most frequently used terms. This may have underestimated the breadth of opioid-related tweets in our sample. Second, we were limited in our ability to validate whether a tweet was indeed about opioids, as it was not possible to identify and query the tweet author about his or her intention. However, research on social media discussions related to cardiovascular mortality [37] and depression [38,39] indicate that these discussions reflect behavioral intentions. Third, filtering out solicitation-related terms and posts with hyperlinks was predicated on the assumption that these tweets reflect illicit opioid sales, which may constitute a unique phenomenon. Indeed, Katsuki et al [15] found that 75.2% of tweets containing URLs linked to an illicit online pharmacy, and Mackey et al [16] found that 90% of online marketing tweets included hyperlinks. Our decision certainly reduced the number of posts in our sample and may have resulted in misclassification. However, given that the overwhelming majority of individuals who misuse opioids report obtaining opioids from friends and family [40], it is likely that this decision had only a small impact on our results. Future research should examine whether tweets that include drug solicitation terms correlate with overdose rates in ways that differ from posts that exclude such terms. Finally, we were limited to correlational analyses without statistical controls, due to insufficient time points needed to run more sophisticated analyses. Our results should be considered preliminary; more research is needed with additional time points and data before making definitive statements.

Limitations notwithstanding, to our knowledge, this study is the first to report that the pattern of opioid-related Twitter posts in North Carolina not only resembles the triphasic nature of the opioid crisis [2], but that tweets mentioning heroin and synthetic opioids also correlate with and predict OODs. Findings suggest that Twitter data should be further evaluated as a novel and timely indicator of opioid overdose mortality, especially for heroin. Twitter use is widespread; of the 68 million Twitter users in the United States, 87% keep their feed public, nearly half of whom report daily usage [41]. Thus, tweets have the potential to serve as a readily available, unique, and real-time data source for surveilling the opioid crisis.

## Abbreviations

CDC: Centers for Disease Control and Prevention
DEA: Drug Enforcement Administration
ICD-10: International Classification of Diseases, 10th revision
OOD: opioid overdose death
WONDER: Wide-ranging Online Data for Epidemiologic Research
